# Novel targeted therapeutics: inhibitors of MDM2, ALK and PARP

**DOI:** 10.1186/1756-8722-4-16

**Published:** 2011-04-20

**Authors:** Yuan Yuan, Yu-Min Liao, Chung-Tsen Hsueh, Hamid R Mirshahidi

**Affiliations:** 1 Division of Medical Oncology and Hematology, Loma Linda University Medical Center, Loma Linda, CA 92354, USA; 2 Department of Internal Medicine, China Medical University Hospital, Taichung, Taiwan, China

## Abstract

We reviewed preclinical data and clinical development of MDM2 (murine double minute 2), ALK (anaplastic lymphoma kinase) and PARP (poly [ADP-ribose] polymerase) inhibitors. MDM2 binds to p53, and promotes degradation of p53 through ubiquitin-proteasome degradation. JNJ-26854165 and RO5045337 are 2 small-molecule inhibitors of MDM2 in clinical development. ALK is a transmembrane protein and a member of the insulin receptor tyrosine kinases. EML4-ALK fusion gene is identified in approximately 3-13% of non-small cell lung cancer (NSCLC). Early-phase clinical studies with Crizotinib, an ALK inhibitor, in NSCLC harboring EML4-ALK have demonstrated promising activity with high response rate and prolonged progression-free survival. PARPs are a family of nuclear enzymes that regulates the repair of DNA single-strand breaks through the base excision repair pathway. Randomized phase II study has shown adding PARP-1 inhibitor BSI-201 to cytotoxic chemotherapy improves clinical outcome in patients with triple-negative breast cancer. Olaparib, another oral small-molecule PARP inhibitor, demonstrated encouraging single-agent activity in patients with advanced breast or ovarian cancer. There are 5 other PARP inhibitors currently under active clinical investigation.

## Introduction

Modern cancer therapeutics has evolved from non-specific cytotoxic agents that affect both normal and cancer cells to targeted therapies and personalized medicine. Targeted therapies are directed at unique molecular signature of cancer cells to produce greater efficacy with less toxicity. The development and use of such therapeutics allow us to practice personalized medicine and improve cancer care. In this review, we summarized preclinical data and clinical development of three important targeted therapeutics: murine double minute 2 (MDM2), anaplastic lymphoma kinase (ALK) and poly [ADP-ribose] polymerase (PARP) inhibitors.

### Murine Double Minute 2

MDM2, also known as HDM2 in human, is a negative regulator of tumor suppressor p53 [1]. MDM2 encodes a 90-kDa protein with a p53 binding domain at the N-terminus, and a RING (really interesting gene) domain at the C-terminus functioning as an E3 ligase responsible for p53 ubiquitylation [2]. When wild-type p53 is activated by various stimuli such as DNA damage, MDM2 binds to p53 at the N-terminus to inhibit the transcriptional activation of p53, and promote the degradation of p53 via ubiquitin-proteasome pathway [3,4]. MDM2 is overexpressed in a variety of human cancers, including melanoma, non-small cell lung cancer (NSCLC), breast cancer, esophageal cancer, leukemia, non-Hodgkin's lymphoma and sarcoma [5]. MDM2 can interfere with p53-mediated apoptosis and growth arrest of tumor, which is the major oncogenic activity of MDM2 [6,7]. Additionally, MDM2 can cause carcinogenesis independent of p53 pathway [8]. In tumor with homozygous mutant p53, loss of MDM2, which mimics the inhibition of the MDM2-p53 interaction, can cause stabilization of mutant p53 and increased incidence of metastasis [9]. Overexpression of MDM2 has been shown to correlate positively with poor prognosis in sarcoma, glioma and acute lymphocytic leukemia [10]. In NSCLC, there have been conflicting results as to whether MDM2 overexpression is associated with worse or better prognosis, but the subset analysis has demonstrated a poor prognostic factor for early-stage NSCLC patients, particularly those with squamous cell histology [11].

### Preclinical development of MDM2 inhibitors

Inhibition of MDM2 can restore p53 activity in cancers containing wild-type p53, leading to anti-tumor effects with apoptosis and growth inhibition [12-14]. Animal studies have shown reactivation of p53 function can lead to the suppression of lymphoma, soft tissue sarcoma, and hepatocellular carcinoma [15-17]. Ventura et al. have designed a reactivatable p53 knockout animal model by a a Cre-loxP-based strategy, which a transcription-translation stop cassette flanked by loxP sites (LSL) is inserted in the first intron of the endogenous wild-type p53 locus leading to silencing of p53 expression. Cells from homozygous p53LSL/LSL mice are functionally equivalent to p53 null (p53-/-) cells, and p53LSL/LSL mice are prone to develop lymphoma and sarcoma. Due to the presence of flanking loxP sites, the stop cassette can be excised by the Cre recombinase, which causes reactivation of p53 expression and regression of autochthonous lymphomas and sarcomas in mice [16].

**Figure 1:**
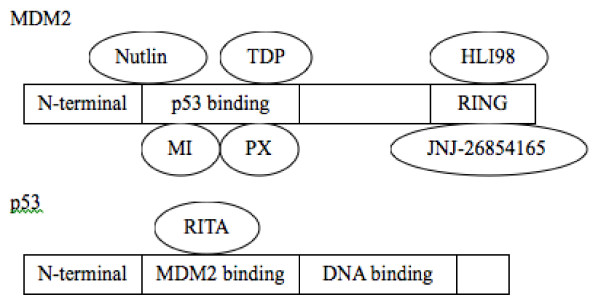
**Schematic representation of the MDM2 and p53 proteins, and the binding areas for small-molecule inhibitors**. Nutlin, cis-imidazoline; TDP, benzodiazepinedione; MI, spiro-oxindoles; PXN, isoquinolinone; HLI98, 5-deazaflavin; JNJ-26854165, tryptamine; RITA, thiophene; RING, really interesting new gene (signature domain of E3 ligase). Binding of either HLI98 or JNJ-26854165 to RING domain of MDM2 can block the interaction of ubiquitinated MDM2-p53 protein complex to the proteasome.

These results have provided an encouraging direction for p53-target therapeutic strategy utilizing inhibition of MDM2. Since the interaction and functional relationship between MDM2 and p53 have been well characterized, small-molecule inhibitors of MDM2 have been developed by high-throughput screening of chemical libraries [18-20]. As shown in table 1, there are three main categories of MDM2 inhibitors: inhibitors of MDM2-p53 interaction by targeting to MDM2, inhibitor of MDM2-p53 interaction by targeting to p53, and inhibitors of MDM2 E3 ubiquitin ligase. The binding sites and mechanism of action for these inhibitors are further illustrated in Figure 1.

**Table 1:** MDM2 inhibitors in development

Chemical series	Therapeutics	Development stage
Inhibitors of MDM2-p53 interaction by targeting to MDM2		

Cis-imidazoline	RO5045337 (RG7112; Nutlin-3)	Phase I: Advanced solid tumors and hematological malignancy

Benzodiazepinedione	TDP521252 & TDP665759	Preclinical

Spiro-oxindoles	MI-219, MI-319 & other MI compounds	Preclinical

Isoquinolinone	PXN727 & PXN822	Preclinical

Inhibitor of MDM2-p53 interaction by targeting to p53		

Thiophene	RITA (NSC 652287)	Preclinical

E3 Ligase Inhibitors		

5-Deazaflavin	HLI98 compounds	Preclinical

Tryptamine	JNJ-26854165	Phase I: Advanced solid tumors

RITA, reactivation of p53 and induction of tumor cell apoptosis

Reference; [23,36,37,130-132,134-139]

Nutlins, consisting of nutlin 1, 2 and 3, analogs of cis-imidazoline, fit in the binding pocket of p53 in MDM2 and inhibit the interaction between MDM2 and p53 [21,22]. Nutlin-3, an analog of the series, has the most potent binding capacity and lowest inhibition concentration, induced p53 levels, and activated p53 transcriptional activity [23]. Nutlin-3 has been shown to exhibit a broad activity against various cancer models with wild-type p53, such as breast, colon, neuroblastoma, mantle cell lymphoma and osteosarcoma [24-27]. Nutlin-3 activates p53 and induces apoptosis and cellular senescence in myeloid and lymphoid leukemic cells {Hasegawa, 2009 #149}. In the absence of functional p53, nutlin-3 interrupts the interaction between p73 and MDM2, and increases p73 transcriptional activity, leading to enhanced apoptosis and growth inhibition of leukemic cell [30].

MDM4 (also known as MDMX), an MDM2 homolog, binds p53 and inhibits p53 activity without causing degradation of p53 degradation [31]. Furthermore, despite the similarity between MDM2 and MDM4, MDM2 inhibitors such as nutlin-3 are far less effective against MDM4 [32]. Small-molecule inhibitor of MDM4 has been developed through a reporter-based drug screening [33]. MDM4 inhibitor not only can activate p53 and induce apoptosis in breast cancer MCF-7 cells, but can also synergize with MDM2 inhibitor for p53 activation and induction of apoptosis.

### Clinical development of MDM2 inhibitors

JNJ-26854165, a novel tryptamine derivative, is an oral MDM2 inhibitor. Pre-clinical studies have shown binding of JNJ-26854165 to RING domain of MDM2 inhibits the interaction of MDM2-p53 complex to the proteasome, and increases p53 level [19]. Furthermore, induction of apoptosis and anti-proliferation independent of p53 in various tumor models including breast cancer, multiple myeloma and leukemia were shown [34-36]. The presence of p53-independent apoptotic activity in addition to p53-mediated apoptosis is regarded as an advantage to prevent the selection of p53 mutant subclones in cancer during treatment of JNJ-26854165. Results for phase I study (clinicaltrial.gov identifier: NCT00676910) using continuously daily oral dosing in patients with advanced solid tumors were presented in 2009 annual meeting of American Society of Clinical Oncology (ASCO) [37]. Forty-seven patients were treated at 11 dose levels, ranging from 4 to 400 mg daily. Treatment was well tolerated with frequent adverse events in grade 1-2: nausea, vomiting, fatigue, anorexia, insomnia, electrolyte imbalance, and mild renal/liver function impairment. No hematological or cardiovascular toxicities were observed. One patient at 300 mg dose level experienced dose-limiting toxicity (DLT) with grade 3 asymptomatic QTc prolongation, which resolved after discontinuation of treatment. Dose escalation was stopped at 400 mg dose level due to 2 out of 3 patients had DLT including one grade 3 skin rash and one grade 3 QTc prolongation. There was no objective response, but 3 patients with prolonged SD including one breast cancer overexpressing human epidermal growth factor receptor 2. Pharmacokinetic study demonstrated linear pharmacokinetics in 20 to 400 mg dose range, with preclinical determined therapeutic concentration achieved at dose level of 300 mg and above. Pharmacodynamic study showed upregulation of p53 in skin, increase of HDM2 levels in tumors, and increase of plasma macrophage inhibitory cytokine-1 (MIC-1) levels in dose-dependent manner. MIC-1, a transforming growth factor-B superfamily cytokine, is induced by p53 activation, and secreted MIC-1 levels can serve as a biomarker for p53 activation [38]. Dose level of 350 mg was used on expanded cohort of patients to confirm maximum tolerated dose, and trial with alternate dosing schedule to minimize QTc prolongation was started with 150 mg twice a day.

RO5045337 (RG7112), an oral formulation of nutlin-3, is currently in phase I studies for patients with advanced solid tumors (NCT00559533), and refractory acute leukemias and chronic lymphocytic leukemia (NCT00623870). Both studies are to determine the maximum tolerated dose and the optimal dosing schedule of RO5045337, administered as monotherapy. Preliminary data has shown acceptable safety profiles with responses seen in patients with liposarcoma, acute myelogenous leukemia and chronic lymphocytic leukemia.

### Anaplastic Lymphoma Kinase

**Figure 2:**
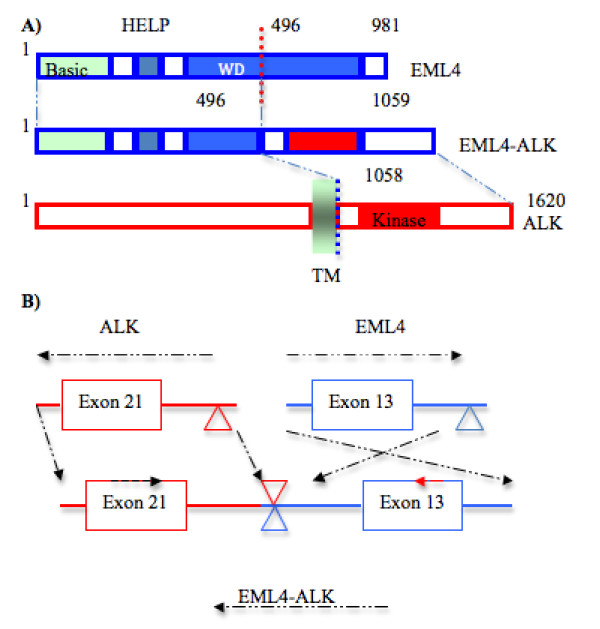
**Schematic representation of the EML-4 and ALK translocation**. A) Fusion of the N-terminal portion of EML4 (comprising the basic region, the HELP domain and part of the WD-repeat region) to the intracellular region of ALK (including the tyrosine kinase domain). TM, transmembrane domain. B) Both the ALK gene and the EML4 gene plot to chromosome 2p, but have opposite orientations. In the NSCLC EML4 is interrupted at a position 3.6 kb downstream of exon 13 and is attached to a position 297 bp upstream of exon 21 of ALK, creating the EML4-ALK (variant 1) fusion gene.

ALK is a 1620 amino acid transmembrane protein, consisting of extracellular domain with amino-terminal signal peptide, intracellular domain with a juxtamembranous segment harboring a binding site for insulin receptor substrate-1, and a carboxy-terminal kinase domain [39]. ALK is a member of the insulin receptor tyrosine kinases, and the physiological function of ALK remains unclear [40]. Translocation of ALK occurs in about 50% of anaplastic large-cell lymphoma (ALCL), and 80% of them have the t (2; 5) chromosomal translocation with NPM-ALK expression [41]. The t (2; 5) translocation generates a fusion protein with carboxy-terminal kinase domain of ALK on chromosome 2, and the amino-terminal portion of nucleophosmin (NPM) on chromosome 5. NPM is the most common fusion partner of ALK, but at least six other fusion partners have been identified. In these fusion proteins, the amino-terminal portion is responsible for protein oligomerization, which activates ALK kinase and downstream signaling such as Akt, STAT3, and extracellular signal-regulated kinase 1 and 2 [42] (Figure 2).

Mutations of ALK have been identified in 6-12% of sporadic neuroblastoma, and preclinical studies have demonstrated these mutations promote ALK kinase activity leading to oncogenic events [43]. It has been postulated that activation of ALK provides oncogenic addiction to tumors harboring activating mutation or translocation of ALK [44]. Knock-down of ALK by small hairpin RNA targeting ALK in NPM-ALK-containing tumor models gives raise to growth inhibition and apoptosis [45]. This indicates inhibition of ALK may be an effective therapeutic strategy for tumors harboring ALK activation.

Echinoderm microtubule-associated protein-like 4 (EML4) is a 120 KDa cytoplasmic protein, which involves in the formation of microtubules and microtubule binding protein [46]. EML4-ALK is a novel fusion gene arising from an inversion on the short arm of chromosome 2 [Inv (2)(p21p23)] that joined exons 1-13 of EML4 to exons 20-29 of ALK [47,48]. Soda et al. identified this fusion gene as a transforming activity in mouse 3T3 fibroblasts from DNA of lung cancer in a Japanese man with a smoking history in 2007 [48]. EML4-ALK fusion protein consists of the complete tyrosine kinase domain of ALK at and the carboxy-terminal, and promoter of the 5' partner gene controls the transcription of the resulting fusion gene. Multiple variants of EML4-ALK have been identified, and all the variants encode the same cytoplasmic portion of ALK but different truncations of EML4 (at exons 2, 6, 13, 14, 15, 18, and 20). In lung cancer the chimeric protein involves ALK fused most commonly but not exclusively to EML4. Other rare fusion partners are TRK-fused gene 11 (TFG 11) and KIF5B (Kinesin heavy chain) [47-51]. ALK gene rearrangements and the resulting fusion proteins in tumor specimen can be identified by fluorescent in situ hybridization (FISH), immunohistochemistry (IHC), and reverse transcription-polymerase chain reaction (RT-PCR).

The presence of EML4-ALK fusion is identified in approximately 3-13% of NSCLC, and mutually exclusive with the presence of epidermal growth factor receptor (EGFR) mutation [48,52-55]. EML4-ALK fusion transcript is not identified in other cancer types such as gastrointestinal and breast cancers [56]. Shaw et al. investigated the clinical features of NSCLC patients harboring EML4-ALK fusion rearrangement [55]. Among 141 patients, they found 19 (13%) patients carried the EML4-ALK rearrangement, 31 (22%) harbored an activating EGFR mutation, and 91 (65%) were wild type for both ALK and EGFR (designated WT/WT). EML4-ALK-positive patients were significantly younger than patients with either EGFR mutation or WT/WT (P < 0.001 and P = 0.005, respectively). EML4-ALK-positive patients were more likely to be men than patients with either EGFR mutation or WT/WT (P = 0.036 and P = 0.039, respectively). EML4-ALK-positive patients were significantly never or light smokers compared with the WT/WT patients (P < 0.001), and did not benefit from treatment with EGFR tyrosine kinase inhibitors (TKIs). Eighteen EML4-ALK-positive patients had adenocarcinoma and one patient had mixed adenosquamous histology. However, patients with EML4-ALK-positive NSCLC did not have exclusively adenocarcinoma histology in two other studies [51,53].

Focusing on the clinical outcome, Shaw et al. examined 477 NSCLC patients, and identified 43 patients (9%) with EML4-ALK rearrangements, 99 patients (21%) with EGFR mutations, and 335 patients (70%) with WT/WT [57]. EML4-ALK-positive patients were significantly younger (median age 54 vs 64 years old, p < 0.001) and more likely to be never or light smokers (90% vs 37%, p < 0.001), compared with WT/WT patients. There was no difference in overall survival (OS) between patients with EML4-ALK fusion and EGFR mutation (1-year OS: 82% vs 81%, p = 0.79); however, both groups demonstrated a longer OS than WT/WT patients (1-year OS 66%, p < 0.001). This data suggests the better outcome in patients with EML4-ALK rearrangement vs. patients with WT/WT may be related to differences in biology, demographic features, and availability of targeted therapies.

### Preclinical development of ALK inhibitors

The development of ALK small-molecule inhibitors has been hampered due to lack of ALK protein structure. Initial testing and development of ALK inhibitors were done with naturally occurring sources such as staurosporine and HSP90 inhibitors, which are not potent and specific inhibitors of ALK [58]. Subsequently, using homology modeling to assist the screening and synthesis, more potent and specific ALK inhibitors have been developed [59]. Although there are multiple partners for the ALK translocation, all the fusion proteins contain the ALK kinase domain and should be susceptible to ALK kinase inhibition. As shown in table 2, there are at least 9 different chemical classes of small-molecule inhibitors of ALK being developed.

**Table 2:** ALK inhibitors in development

Chemical series	Therapeutics	Development stage
Aminopyridine	PF-2341066 (crizotinib)	Phase II/III: NSCLC; phase I/II: advanced solid tumors, neuroblastoma, and ALCL

Diaminopyrimidine	CEP-28122	Preclinical IND application expected

Structure undisclosed	AP-26113	Preclinical IND application expected in 2011

Structure undisclosed	X-276	Preclinical

Pyridoisoquinoline	F91873 and F91874	Preclinical

Pyrrolopyrazole	PHA-E429	Preclinical

Indolocarbazole	CEP-14083 and CEP-14513	Preclinical No further development

Pyrrolopyrimidine	GSK1838705A	Preclinical No further development

Dianilinopyrimidine	NVP-TAE684	Preclinical No further development

NSCLC, non-small cell lung cancer; ALCL, anaplastic large cell lymphoma

Reference [60,61,64,65,140-150]

PF-2341066 (Crizotinib), derivative of aminopyridine, was initially developed as a potent, orally bioavailable, ATP-competitive small-molecule inhibitor of c-MET and hepatocyte growth factor receptor [60]. Further investigation has indicated Crizotinib is a potent inhibitor of ALK as well, and half maximal inhibitory concentration (IC_50_) for either c-MET or ALK overexpressing cell line is ~20 nM. Crizotinib suppresses the proliferation of ALCL cell line with ALK activation, but not in ALCL cell lines without ALK activation. Crizotinib inhibits phosphorylation of ALK, and causes complete regression of ALCL harboring NPM-ALK fusion in xenograft model [61]. Crizotinib also inhibits the proliferation in NSCLC (such as H3122) and neuroblastoma cell lines harboring ALK activation [62]. Experiments using NCI-H441 NSCLC xenografts showed a 43% decrease in mean tumor volume, with 3 of 11 mice exhibiting a >30% decrease in tumor mass and 3 animals with no evidence of tumor at the end of the 38-day crizotinib treatment [60]. Crizotinib is currently undergoing active clinical investigation in NSCLC. Additionally, phase I/II study is conducted in patients with advanced malignancy such as ALCL or neuroblastoma (NCT00939770).

Second-generation ALK inhibitors such as AP-26113 and X-276 are considered more potent and selective inhibitors of ALK than crizotinib. AP-26113, an orally bioavailable inhibitor of ALK with undisclosed structure, is developed by Ariad [63]. During preclinical investigation, AP-26113 has been shown to inhibit not only the wild-type ALK but also mutant forms of ALK, which are resistant to the first-generation ALK inhibitor such as crizotinib. Further studies have demonstrated AP-26113 is at least 10-fold more potent and selective in ALK inhibition than crizotinib [64,65].

### Clinical development of ALK inhibitors

In 2009 annual meeting of ASCO, Kwat et al. reported the results of phase I dose escalation study and expanded phase II study of crizotinib [66]. Thirty-seven patients with advanced solid tumors including 3 NSCLC patients were enrolled in phase I study. The maximum tolerated dose of crizotinib was 250 mg orally twice a day, and 2 fatigue DLT were noted in the next dose level at 300 mg twice a day. The major side effects were fatigue, nausea, vomiting and diarrhea; but were manageable and reversible. There was 1 partial response (PR) in a sarcoma patient with ALK rearrangement. Additionally, a dramatic clinical response was observed in a NSCLC patient harboring EML4-ALK rearrangement. Therefore, an expanded phase II study using 250 mg of crizotinib twice a day was conducted in 27 NSCLC patients harboring EML4-ALK tumor determined by FISH. In the first 19 evaluable patients, there were 17 patients with adenocarcinoma (90%) and 14 non-smokers (74%). Overall response rate (RR) was 53%, and disease control rate (DCR; complete response [CR]/PR/stable disease [SD]) was 79% at 8 weeks. Only 4 patients (21%) progressed after 8 weeks of treatment, despite more than 60% of patients received 2 or more lines of treatment prior to entering this study.

Bang et al. presented the follow up results on the expanded phase II study of crizotinib in NSCLC patients with EML4-ALK rearrangement in 2010 annual meeting of ASCO [67]. Eighty-two patients were evaluable, with 96% adenocarcinoma, 76% never-smokers and ~95% having prior treatment. Overall RR was 57%, with estimated 6-month progression-free survival (PFS) rate of 72%, and DCR of 87% at 8 weeks. The median progression-free survival was not yet mature, and the median duration of treatment was 25.5 weeks. Radiological responses typically were observed at the first or second restaging CT scan. Main side effects were nausea, diarrhea and visual disturbance on light/dark accommodation without abnormality on eye examination. The results of this phase II study have been recently published [68].

Based on these encouraging results, a randomized phase III trial comparing crizotinib to standard second-line cytotoxic chemotherapy docetaxel and pemetrexed in patients with ALK-positive NSCLC has now commenced (NCT00932893). The combination of erlotinib and crizotinib is also being tested in patients who failed prior chemotherapies regardless of EML4/ALK translocation status (NCT00965731). A phase III study to evaluate crizotinib as first line therapy in EML4-ALK translocation patients compare to standard platinum based chemotherapy is underway (NCT01154140).

### Poly ADP-Ribose Polymerases

PARPs are a family of nuclear enzymes that regulates the repair of DNA single-strand breaks (SSBs) through the base-excision repair (BER) pathway [69]. Upon DNA damage, PARP cleaves nicotinamide adenine dinucleotide (NAD^+^) to generate poly (ADP-ribose) (PAR) polymers, which are added onto DNA, histones, DNA repair proteins and PARP [70,71]. These hetero- and auto-modification processes mediated by PARP lead to recruitment of repair machinery to facilitate BER process. Among the 17 members of PARP, PARP-1 and PARP-2 are the only members known to be activated by DNA damage and may compensate for each other [72]. PARP-1 is best characterized and responsible for most if not all the DNA-damage-dependent PAR synthesis; exhibits with N-terminal DNA-binding domain, central auto-modification domain, and C-terminal catalytic domain, which is the "signature" for PARP family. Although lacks of central auto-modification domain, PARP-2 shares ~70% homology of catalytic domain as PARP-1, and provides residual PARP activity (~15%) in the absence of PARP-1 [73]. The physiological functions of PARP-1 and PARP-2 have been further explored in knockout models [74]. Double PARP-1 and PARP-2 knockout mice are lethal at the embryonic stage. Knockout of either PARP-1 or PARP-2 results in increased genomic instability by accumulation of DNA SSBs, and causes hypersensitivity to ionizing radiation and alkylating agents. Additionally, PARP-1 also plays important roles in cellular responses to ischemia, inflammation and necrosis.

**Figure 3:**
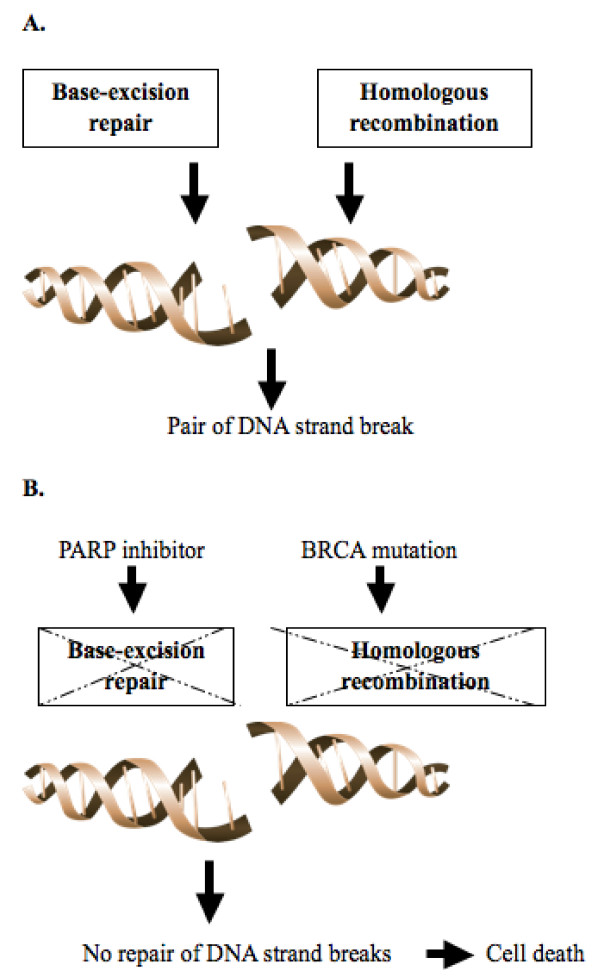
**Schematic representation of PARP and BRCA mediated DNA repair in cells without exposure to PARP inhibitor and BRCA mutation (A), and synthetic lethality in cells with BRCA mutation exposing to PARP inhibitor (B)**.

Targeting the PARP-mediated DNA repair pathway is a promising therapeutic approach for potentiating the effects of chemotherapy and radiation therapy and overcoming drug resistance [75]. However, the most exciting use of PARP inhibitors may be utilizing a phenomenon called synthetic lethality [76]. Synthetic lethality is a cellular condition in which simultaneous loss of two nonessential mutations results in cell death, which dose not occur if either gene products is present and functional [77]. Tumors with DNA repair defects, such as those arising from patients with BRCA mutations were found to be more sensitive to PARP inhibition due to synthetic lethality. The BRCA1 and BRCA2 gene encodes large proteins that coordinate the homologous recombination repair double strand breaks (DSBs) pathway [78]. Since BRCA1/2-mutated tumors cannot utilize homologous recombination to repair DSBs, exposing these cells to PARP inhibitor, which shuts down BER rescue pathway, will lead to accumulation of DNA damage, genomic instability and cell death (Figure 3).

### Preclinical development of PARP inhibitors

Inhibition of PARP has been developed in the laboratory for more than 30 years, with analogues mimicking nicotinamide component of NAD^+ ^for binding to catalytic site of PARP [70,79]. Preclinical data reporting efficacy of PARP inhibitors in a *BRCA *mutated population was initially reported in 2005 [80,81]. Bryant et al. revealed that low concentrations of PARP inhibitors produced cytotoxicity on BRCA2-deficient cell lines with defects in homologous recombination, but not in cell lines with intact homologous recombination. When BRCA2 function was restored in these cell lines, the cells were no longer subject to inhibition of PARP. In other breast cancer cell lines such as MCF-7 and MDA-MB-231, similar sensitivity to PARP inhibition was observed when BRCA2 was depleted. Similarly, Farmer et al. demonstrated that PARP inhibitors NU1025 and AG14361 were highly cytotoxic in BRCA2-deficient VC-8 cells [80]. Additionally, cell death increased when BRCA1/2 deficient cells were transfected with small interfering RNA targeting *PARP-1*. Enhanced sensitivity to PARP inhibition in BRCA-deficient cells was observed when DNA-damaging agents were added in vitro. These preclinical data serve as proof-of-concept for synthetic lethality in BRCA-deficient cell lines and provide important rationale for studying PARP inhibitors in patients with BRCA1/2-asssociated breast and ovarian cancer.

Further investigations have identified triple-negative breast cancer (TNBC, breast cancer without over-expression of estrogen, progesterone and HER2-neu receptors, accounts for about 15 percent of all breast cancers) and sporadic serous ovarian cancer without mutations of BRCA1/2 but exhibit properties of BRCA1- or BRCA2-deficient cells, known as "BRCAness" [82]. BRCAness cancers have defects in homologous recombination due to dysfunctional BRCA1/2 from epigenetic modification, and/or deficiency in proteins involved in homologous recombination repair pathways, such as RAD51, RAD54, DSS1, RPA1, ATM, CHK2 and PTEN [83-85]. Preclinical studies have shown BRCAness cancer cells are more sensitive to PARP inhibition especially in the presence of DNA-damaging agents such as cisplatin, vs. non-BRCAness [86]. These important findings have further expanded the therapeutic application of PARP inhibitors in cancers with acquired defect in homologous recombination other than germline BRCA mutations.

As shown in table 3, there are currently 9 different PARP inhibitors at different stages of clinical development, and at least 3 highly selective PARP inhibitors in preclinical development. Because both PARP-1 and PARP-2 share high degree of homology in catalytic domain, most of the PARP inhibitors under clinical development do not have significant differential activity against either PARP-1 or PARP-2 [69]. Using x-ray crystal structure and homology modeling, highly selective inhibitors against either PARP-1 or PARP-2 have been successfully developed [87-89]. Over-activation of PARP-1 due to DNA damage from ischemic event is responsible for post-ischemic cell death in neurons and myocardial cells, and PARP-1 knockout mice are more resistant to the damage from ischemic insults [90,91]. PARP inhibitors such as INO-1001 and MP-124 have been studied in animal models and clinical settings as neuroprotectant and cardiac protectant during ischemic insults [92-94].

**Table 3:** PARP inhibitors in development

Chemical series	Therapeutics	Development stage
Benzamide	BSI-201 (iniparib)	Phase III Gemcitabine and carboplatin ± BSI-201 in breast and lung cancers; phase I/II: single agent or combination with chemotherapy in various cancer types including glioma and ovarian cancer

Phthalazinone	AZD2281 (olaparib)	Phase I/II Single agent or combination with chemotherapy in various cancer types including breast, ovarian and colorectal cancers

Tricyclic indole	AG-014699 (PF-01367338)	Phase II Single agent in BRCA-associated breast or ovarian cancer; Phase I: combination with chemotherapy in advanced solid tumors

Benzimidazole	ABT-888 (Veliparib)	Phase II Combination with chemotherapy in various cancer types including breast cancer, colorectal cancer, glioblastoma multiforme and melanoma; phase I: combination with radiation

Indazole	MK-4827	Phase I Single agent; combination with carboplatin-containing regimens

Pyrrolocarbazole	CEP-9722	Phase I Combination with temozolomide in advanced solid tumors

Phthalazinone	E7016 (GPI-21016)	Phase I Combination with temozolomide in advanced solid tumors

Isoindolinone	INO-1001	Phase I Combination with temozolomide in melanoma (completed) without further investigation in oncology; phase II in cardiovascular disease

Structure undisclosed	MP-124	Phase I in acute ischemic stroke

Structure undisclosed	LT-00673	Preclinical

Structure undisclosed	NMS-P118	Preclinical

Structure undisclosed	XAV939	Preclinical, highly selective against PARP-5 (tankyrase)

Reference; [94,96,97,100,101,103,106,108,115-117,119,122-125,151-156]

PARP-5a and PARP-5b, also known as tankyrase 1 and tankyrase 2, are involved in telomere metabolism and Wnt/β-catenin signaling [69]. Moreover, tankyrase inhibition imposes selective lethality on BRCA deficient cell lines [95]. XAV939, a small molecule which suppresses β-catenin-mediated transcription by stabiling axin and degrading β-catenin, is found to inhibit tankyrases [96]. Molecule like XAV939 can be used to target cancers harboring BRCA (such as breast cancer) and/or dysregulated Wnt-β-catenin signaling (such as colorectal cancer) without affecting PARP-1.

### Clinical development of PARP inhibitors

Seven PARP inhibitors are currently in clinical development in oncology. Most of phase I studies have used pharmacodynamic analysis of PARP-1 activity in peripheral blood mononuclear cells (PBMCs) to determine the optimal PARP inhibitory dose. There are 2 main investigational approaches: single-agent study in BRCA-associated and BRCAness cancers; combination study with DNA-damage agent and/or radiation. BSI- 201 (Sanofi-Aventis) is currently in a phase III trial for TNBC in combination with gemcitabine and carboplatin. AZD2281 (Astra-Zeneca), AG-014966 (Pfizer) and ABT-888 (Abott), are in phase II clinical trials as single agent or in combination with chemotherapy. MK-4827 (Merck), CEP-9722 (Cephalon) and E7016 (Eisai) are in phase I clinical trials. INO-1001 (Inotek) is no longer in clinical development after completion of a phase IB study in combination with temozolomide in patients with advanced melanoma [97], and there is no updated information available on this compound [98,99].

### BSI-201 (Iniparib)

BSI-201 is different from other PARP inhibitors, due to drug discovery from interacting with DNA binding domain of PARP-1 instead of catalytic site of PARP [100]. By disrupting the binding between PARP-1 and DNA, BSI-201, a noncompetitive PARP-1 inhibitor, attenuates PARP-1 activation. Phase I study of BSI-201 in advanced solid tumors has demonstrated good tolerability without an identified MTD with dose levels ranging from 0.5 mg/kg to 8.0 mg/kg IV twice weekly. The most common adverse event was gastrointestinal toxicity (39%). At dose level of 2.8 mg/kg, PARP was inhibited in PBMCs by greater than 50% after a single dose, with greater inhibition observed (80% or more) after multiple dosing [101]. A phase IB study combining BSI-201 with various chemotherapeutic agents such topotecan, gemcitabine, temozolomide, and carboplatin/paclitaxel in patients with advanced solid tumors has shown acceptable safety profiles at doses levels ranging from 1.1 to 8.0 mg/kg iv twice a week [102]. Significant PARP inhibition was again noted at dose levels of 2.8 mg/kg or higher. Of 55 patients in this study, there were one CR (ovarian cancer), 5 PR (2 breast cancer, and 3 other cancer types) and 19 SD. In 2009, O'Shaughnessy et al. presented the results of a randomized phase II study comparing gemcitabine plus carboplatin with or without BSI-201 (5.6 mg/kg; iv; biweekly n days 1, 4, 8, and 11 every 21 days) in patients with TNBC [103]. The addition of BSI-201 improved RR from 16% to 48% (p = 0.002), and DCR from 21% to 62%. Median PFS was improved from 3.3 to 6.9 months (hazard ratio [HR] 0.34, p < 0.001). Final result of this phase II study was reported at 2009 San Antonio Breast Cancer Symposium with overall survival was improved from 7.7 to 12.2 month (HR 0.5, p = 0.005) [104]. It's noted that no significant difference in myelotoxicity was seen between the two treatment arms. An updated analysis reported at 2010 European Society for Medical Oncology meeting showed PFS was improved from 3.6 months to 5.9 months (HR 0.59) and DCR was improved from 33.9% to 55.7% (p = 0.015), median overall survival benefit remain similar (7.7 months vs. 12.3 months, HR 0.57). A randomized phase III study comparing gemcitabine plus carboplatin with or without BSI-201 in patients with TNBC is currently underway (NCT00938652). Similar treatment design is used for an ongoing phase III study in patients with stage IV squamous cell lung cancer (NCT01082549). BSI-201 is also currently being evaluated as single agent or combination with chemotherapy in phase I/II studies in various cancer types including glioma and ovarian cancer.

### AZD2281 (Olaparib)

Fong et al. reported the results of phase I study of olaparib, which is an oral small-molecule PARP inhibitor [105,106]. The frequently occurred toxicities were nausea, vomiting, diarrhea, and fatigue. Maximum tolerated dose (MTD) was identified at 400 mg twice daily, with grade 3 fatigue and mood alteration DLT noted in one of eight patients at this dose level. Grade 4 thrombocytopenia and grade 3 somnolence occurred in two of five patients receiving 600 mg twice daily. In a group of 19 patients with breast, ovarian or prostate caners with known *BRCA *mutation, RR of 47% and DCR of 63% were observed without profound difference in toxicity profiles in comparison with non-BRCA mutated patients [106]. The subsequent phase II study in 27 breast cancer patients with BRCA mutation (18 BRCA1 deficient and 9 BRCA2 deficient) showed RR of 41% and median PFS of 5.7 months [107]. The pooled analysis of 50 ovarian cancer patients with BRCA1/2-mutation treated on phase I and II studies (11 on phase I, and 39 on phase phase II receiving olaparib 200 mg twice daily) showed RR of 40% and DCR of 46%, predominately in platinum-sensitive group [108].

Two subsequent Phase II studies evaluating olaparib in previously treated BRCA1/2-mutated breast cancer and ovarian cancer patients were recently reported [104,109,110]. In both studies, patients were treated with either 100 mg or 400 mg of olaparib twice daily. Fifty-seven ovarian cancer patients and 54 breast cancer patients were studies respectively. Overall RR in the ovarian cancer study was 33% in the high-dose group and 13% in the low-dose group. Overall RR in the breast cancer study was 41% in the high-dose group and 22% in the low-dose group.

Interestingly, reported in 2010 annual meeting of ASCO, a provocative phase II study of olaparib showed promising results for women with high-grade serous ovarian cancer regardless of BRCA mutation status [111]. Patients with advanced breast or ovarian cancer were treated with single agent olaparib 400 mg twice daily continuously for 28-day cycle. Of 64 women with ovarian cancer in the study, the overall RR was 41.2% and 23.9%, respectively, for patients with and without BRCA mutations. However, no response was seen in the 24 patients with TNBC treated with olaparib. This is the first single-agent trial demonstrating promising activity of olaparib in high-grade non-BRCA mutated sporadic serous ovarian caner. The mechanism could be attributed by underlying DNA repair abnormalities, which may lead to "BRCAness"[82,112].

Combinations of olaparib and chemotherapy agents have been explored. Myelosuppresion decreases tolerability when combine olaparib with chemotherapy agents [113]. Dent et al. reported a phase I/II study of olaparib in combination with weekly paclitaxel as first or second-line treatment in patients with metastatic TNBC [114]. Olaparib 200 mg twice daily was given continuously with paclitaxel 90 mg/m^2 ^weekly for 3 of 4 weeks. Toxicity included 58% neutropenia, 63% diarrhea, 58% nausea, and 53% fatigue, and most were grade 1-2 except neutropenia. Of 19 patients treated in two cohorts, RR of 33 to 40% and median PFS of 5.2 to 6.3 months were observed.

### AG-014699 (PF-01367338)

AG-014699, an intravenous PARP inhibitor, was studied in combination with temozolomide in advanced solid tumors [115]. PARP inhibitory dose was decided at 12 mg/m^2 ^IV daily for 5 days every 4 weeks based on 74% to 97% inhibition of peripheral blood lymphocyte PARP activity. Mean tumor PARP inhibition at 5 h was 92% (range, 46-97%). No significant toxicity was seen from AG-014699 alone, and AG-014699 showed linear pharmacokinetics without interaction with temozolomide. A phase II study with this combination as 1^st ^line treatment of 40 patients with metastatic melanoma showed RR of 10% and SD of 10%, with significant bone marrow suppression being the major toxicity [116]. Currently, this compound is in phase II study as single agent in patients with advanced BRCA1/2 mutated breast or ovarian cancer (NCT00664781), and in phase I study in combination with cytotoxic agents in patients with advanced solid tumor (NCT01009190).

### ABT-888 (Veliparib)

ABT-888 is an oral PARP inhibitor. Preclinical studies in breast cancer, melanoma and glioma models demonstrated that ABT-888 potentates the chemotherapy effect of a number of agents including temozolomide, platinum, and irinotecan, as well as radiation [117]. Tan et al. reported the preliminary result of a phase I trial of ABT-888 in combination with cyclophosphamide in patients with advanced solid tumors [118]. ABT-888 50 mg twice daily can be safely combined with cyclophosphamide 750 mg/m^2^. ABT-888 does not alter the pharmacokinetics of cyclophosphamide. This study is still ongoing to determine the MTD of ABT-888 and cyclophosphamide combination.

A phase I study of ABT-888 in combination with metronomic cyclophosphamide revealed activity in BRCA mutated ovarian cancer and TNBC [119]. A phase II trial of ABT-888 40 mg twice daily on days 1 to 7 in combination with temozolomide 150 mg/m^2^, days 1-5 on a 28 days cycles for metastatic breast cancer was well tolerated [120]. However, activity was limited to BRCA mutation carriers. Of 8 patients with BRCA1/2 mutation, 37.5% RR and 62.5% DCR were observed. Medial PFS was 5.5 months in BRCA mutation carriers vs. 1.8 months in non-carriers. This study calls into question of "BRCAness" for at least this PARP inhibitor. ABT-888 is currently being evaluated in many phase I/II studies in combination with chemotherapy or radiation in patients with advanced solid tumors.

### MK-4827

MK-4827 is an orally bioavailable PARP inhibitor. This compound displays potent PARP-1 and PARP-2 inhibition, and inhibits proliferation of breast cancer cells with mutant BRCA-1 and BRCA-2 with IC_50 _in the range of 10-100 nM [121]. Sandhu et al reported phase I result of MK-4827 in 59 patients with advanced solid tumors in 2010 annual meeting of ASCO [122]. MTD was identified at 300 mg daily with common toxicities in nausea/vomiting, fatigue and thrombocytopenia. Two out of six patients on 400 mg daily experienced DLT with grade 4 thrombocytopenia was seen in 2 out of 6 patients received 400 mg daily. Antitumor activity was observed in patients with BRCA-deficient cancers (32% PR in 19 patients with ovarian cancer, and 50% PR in 4 patients with breast cancer). Additionally, PR was seen in 1 patient with sporadic platinum-sensitive ovarian cancer. These findings have shown good tolerability and promising antitumor activity of MK-4827 in both BRCA-deficient and sporadic cancers. Phase I study in expanded cohorts with sporadic ovarian and prostate cancers is currently underway (NCT00749502). Phase IB dose escalation study of MK-4827 in combination with carboplatin, carboplatin/paclitaxel or carboplatin/doxil in patients with advanced solid tumors has also been activated (NCT01110603).

### CEP-9722

Preclinical studies have shown CEP-9722 enhances cellular sensitivity toward temozolomide, irinotecan and radiation in various cancer types such as glioblastoma, colon cancer, neuroblastoma, and rhabdomyosarcoma [123]. CEP-9722 is currently undergoing phase I trial as single agent and in combination with temozolomide in advanced solid tumor (NCT00920595).

### E7016

E7016 (previously known as GPI-21016) is an orally bioavailable PARP inhibitor. In murine leukemia model, E7016 enhances cisplatin-induced cytotoxicity and ameliorated cisplatin-induced neuropathy at the same time, suggesting a role to improve the therapeutic margin of certain cytotoxic agent [124]. Further study in human glioblastoma cell line and xenograft, E7016 enhances tumor radiosensitivity, and synergizes with combination treatment of temozolomide and radiation [125]. There is an ongoing phase I study with dose escalation of E7016 in combination with temozolomide in patients with advanced solid tumors and gliomas (NCT01127178).

## Summary

We reviewed preclinical data and clinical development of MDM2, ALK and PARP inhibitors. Cancer treatment is entering an exciting chapter in targeted therapies and personalized medicine due to the advance of molecular biology and medicinal chemistry. Most likely several compounds from this review will be approved for clinical use in the years to come. Many questions remain to be answered: (1) what are the long-term safety and toxicities of these inhibitors (2) how to use biomarkers to select patients who will benefit most from these inhibitors (3) how to combine these targeted therapies with cytotoxic agents or other treatment modality such as radiation modality in selected patient population?

More than 50 percent of human tumors contain a mutation or deletion of the p53 gene. Mutation of p53 can confer dominant-negative or gain-of-function effects [126]. Dominant-negative effects lead to suppression of wild-type p53 protein in heterozygous mutant cells and a p53 null phenotype; gain-of-function effects result in promotion of tumor development. There have been concerns on the exposure of MDM2 inhibitors to tumors with mutant p53, which potentially can have deleterious effects due to stabilization of mutant p53 [127].

Cautions need to be taken with long-term use of PARP inhibitors. PARP-1 serves important roles in other cellular function such as transcription regulation, initiation of a unique cell death pathway, restarting stalled replication forks, and modulation of cellular responses to ischemia, inflammation and necrosis [128]. Previous studies indicated that genetic ablation of PARP-1 in combination with p53 knockout increased cancer incidence in mice [129]. This raises concern that long-term PAPR-1 inhibition could potentially increase the risk of secondary malignancies.

## Competing interests

The authors declare that they have no competing interests.

## Authors' contributions

HRM and CTH designed the paper. YY, YML, CTH and HRM wrote the paper. All authors read and approved the final manuscript.
